# Wolf in Sheep’s Clothing? Violent, Abusive, and Harmful Behavior by the Older Person Toward Their Family Caregivers: A Qualitative Study

**DOI:** 10.1177/08862605241263589

**Published:** 2024-07-27

**Authors:** Sofia von Humboldt, Namrah Ilyas, Isabel Leal

**Affiliations:** 1 William James Center for Research, ISPA—Instituto Universitário, Lisbon, Portugal; 2 Center for Clinical Psychology, University of the Punjab, Lahore, Pakistan

**Keywords:** carers, family caregivers, mental health, older adults, violent, abusive and harmful behavior

## Abstract

Violent, abusive, and harmful behavior enacted by older adults upon their caregivers represents a distressing and frequently disregarded facet within the domain of caregiving. This qualitative study aims to (a) explore family caregivers’ experiences of violent, abusive, and harmful behavior by the older person and (b) explore how violent, abusive, and harmful behavior by the older person affects family caregivers’ mental health. This qualitative study encompassed 393 participants, with a diverse age range spanning from 40 to 72 years. All the interviews went through the process of content analysis. For the first objective, findings indicated six emerging themes: Frequent and extreme verbal violence (77.3%); feeling manipulated and controlled by older adults (74.7%); experiencing unpredictable illegal circumstances provoked by older adults (62.1%); experiencing damaging financial issues provoked by older adults (43.1%); experiencing physical violence (34.2.%); and experiencing sexual violence (31.1%). The second objective highlighted four themes: depression and anxiety (89.9 %), anger (81.2%), feeling morally isolated (78.3%), and emotional outbursts (65.1%). Brazilian participants mainly experienced frequent and extreme verbal violence (62.4%). Moreover, depression and anxiety were mainly verbalized by English participants (84.3%). These findings underscore the significant toll that older individuals’ violent, abusive, and harmful behavior can have on the mental well-being of family caregivers. This study sheds light on the complex experiences faced by family caregivers and emphasizes the urgent need for targeted interventions to foster healthier caregiving environments. Older individuals’ violent, abusive, and harmful behavior toward their caregivers has received limited attention in research and public discourse. The findings of this study call attention to the pressing need of addressing this issue, given its detrimental impact on the mental health of family carers. Recognizing the significance of this topic demands a comprehensive and targeted approach to ensure the well-being and safety of caregivers and older adults.

## Introduction

Globally, the average mortality age has climbed gradually over the past 50 years, and the number of older adults compared to younger adults and children keeps growing yearly (World Health Organization, 2015). Additionally, long-term, co-occurring, and complicated illnesses are becoming a more prevalent aspect of poor health in later life (Pinquart & Sörensen, 2011; von Humboldt & Leal, 2015; von Humboldt, Leal, & Pimenta, 2013; von Humboldt et al., 2014a, 2021). Within this framework, there has been a notable increase in the number of families assuming the role of caregivers, resulting in family or informal carers constituting the largest demographic of individuals providing care for older adults. Older adults typically refer to individuals aged 65 and above, although this categorization may not be universally consistent (Pinquart & Sörensen, 2011).

In the context of this article, abuse, violence, or severe aggression is operationally defined as the manifestation of physical aggression or behavior involving actual, attempted, or threatened harmful physical contact toward caregivers by care recipients. This encompasses acts of assault that may lead to significant or minor bodily harm or no harm. Additionally, it encompasses credible threats or acts of aggression directed toward property, eliciting a sense of fear or danger within the caregiver (Wharton & Ford, 2014). Simply, when caregivers experience frequent and extremely verbal, physical, and sexual violence; feel manipulated and controlled by their dependent family member for whom they care because of an illness, disability, or old age; and when families live in unpredictable, often chaotic circumstances in which the dynamics of power, love, and duty are complex and closely intertwined, it is characterized as caregivers’ abuse (von Humboldt & Leal, 2014a; von Humboldt, Leal, Pimenta, & Niculescu, 2013; von Humboldt, Leal, Santos, & Niculescu, 2013; von Humboldt, Low, & Leal, 2020, 2022; von Humboldt, Ribeiro-Gonçalves, & Leal, 2022).

Family caregivers encounter numerous challenges while caring for older relatives at home, including difficulties with communication, financial burdens, illegal transactions, theft, and managing medication and transportation. Additionally, effective communication is often hindered when the older relative constantly displays unpredictable behavior (Hailu et al., 2024). Compounding these challenges, older adults represent a particularly vulnerable population for financial exploitation due to age-related changes in cognition and socioemotional functioning, which can further affect caregivers (Ebner et al., 2023).

There are increasing reports of violence against caregivers in an expanding body of literature. This phenomenon is particularly prevalent among caregivers who assist individuals with chronic diseases, including mental health disorders (Pinyopornpanish et al., 2022). Nevertheless, the current body of scholarly work on caregivers experiencing abusive behavior lacks consensus regarding a unified term to describe this phenomenon comprehensively. Intense hostility and violence directed against caregivers by older adults present difficulties in delineating abusive behavior, mainly due to the prevalence of aging spouses or adult children as older people’s caretakers (Pinquart & Sörensen, 2011; Pinyopornpanish et al., 2022). Relational ties often blur the lines, and this familial dynamic can complicate the identification of abuse. Moreover, Isham et al. (2019) highlighted the influence of individuals’ lifelong experiences on their perceptions and responses to the illness and behaviors of family members, impacting their interpretation and handling of abusive behavior by carers. Additionally, decisions to remain in violent relationships are influenced by moral responsibility and worries about starting over after decades of marriage (Policastro & Finn, 2015). These factors complicate the clear identification and categorization of carer’s abuse toward caregivers in the family setting.

According to Phillips et al. (2000), the occurrence of abuse inflicted by older adults upon caregivers deviates from the usual conceptualization of abuse in old age. Consequently, this form of abuse tends to receive less recognition in terms of its significance and prevalence despite legitimate concerns regarding the physical and emotional risks involved.

The relationship between family caregivers and care recipients in explaining abuse/neglect is complex (Steinmetz, 1988). Despite the vast research studies mapping the incidence, prevalence, and consequences of caregivers experiencing abusive behavior (Pillemer et al., 2016), there has been much less consideration of what happens when it is the family caregiver who is adversely affected by the violent or abusive behavior of the older person for whom they care (Isham et al., 2019). Erosa et al. (2010) mentioned in their research study that among 147 caregivers, 51% reported abuse by their care recipient. Moreover, a study by Wilks et al. (2011) revealed that carers of individuals with Alzheimer’s disease (AD) reported a low occurrence of hostility exhibited by the care recipient. However, as the disease progressed, the frequency of aggressive behavior displayed by older adults rose. In their research study, Hansen et al. (2020) mentioned that over one-third of their 642 participants were abused by dementia-suffering care recipients.

Despite the scarce literature, a few research studies still manage to explore this sensitive and complex topic from different dimensions. Moreover, this is not a new concept and area of research. Since the late 1980s, researchers have been investigating care dependency to contribute to the existing literature. Care dependency can be defined as a subjective, secondary need for support in the domain of care to compensate for a self-care deficit (Boggatz et al., 2007). According to Steinmetz (1988), dependency-focused explanations (including caregiver burden/stress) recognize that the dynamics, including abuse by the care recipient, within a caregiving relationship can lead to the caregiver acting out. Concerning this, the complex caregiving relationship can lead to emotional outbursts of caregivers as a response to harmful behavior (Steinmetz, 1988) and to victim blaming (i.e., the older adult caused someone to victimize them), which influenced researchers to focus on examining characteristics of caregivers and other potential offenders (Boggatz et al., 2007; Steinmetz, 1988). Although all these research studies touch on the psychological consequences of caregivers experiencing abusive behavior, there is no research study in the authors’ knowledge that examines the mental health of caregivers experiencing the abusive behavior of the older care recipient.

Literature also highlighted the influence of past victimization, abuse, and long-term violence within families (e.g., child maltreatment and intimate partner violence) on the experiences of caregivers and care recipients (Phillips et al., 2000; Wang et al., 2019; von Humboldt Leal, Laneiro, et al., 2013; von Humboldt & Leal, 2014b, 2017; von Humboldt Leal, Laneiro, et al. (2013), von Humboldt & Leal (2014b), (2017) found that caregivers who face long-term violence, isolation, and past trauma exhibited higher risk factors associated with elder mistreatment. These individuals showed increased caregiver stress and burden levels and reduced social support and resilience. Moreover, their childhood experiences of trauma and violence were reported as more severe. Additionally, family caregivers may have limited training and education in recognizing and addressing distress compared to professional caregivers, and they might also be more susceptible to experiencing cumulative and prolonged stress, often termed as “caregiver burden” (Isham et al., 2019).

Acknowledging that the care relationship is a personal and intimate experience is crucial. Various factors influence it, such as the nature of relationships, spiritual beliefs, and cultural backgrounds. Within these contexts, family members manage their roles and obligations to meet each other’s needs (Kittay, 2011). Therefore, understanding the complex and specific nature of caregiving, encompassing both providing and receiving care, is imperative. Many caregivers perceive their role as fundamentally relational, imbued with personal and societal significance (Larkin et al., 2019).

The primary focus of this study was not solely on categorizing behavior as symptoms of illness or acts of abuse, as commonly found in existing literature (Isham et al., 2019). Moreover, our main objective is to comprehend cross-national family caregivers’ experiences when faced with violent, abusive, and damaging behavior exhibited by older individuals under their care. The second purpose is to investigate the complex and subjective effects of violent, abusive, and damaging conduct exhibited by older individuals on the mental health of family caregivers. In this sense, this research aims to ascertain the psychological impact of caregiver maltreatment, encompassing emotions.

## Method

### Recruitment and Sampling

To recruit the participants for this multicultural study, recruitment efforts spanned three distinct countries: Brazil, the United Kingdom, and Portugal. Initially, 400 individuals of diverse nationalities (Brazilian, English, and Portuguese) were contacted with the intent of their participation. However, seven participants were excluded due to data unavailability or incomplete information. The final non-probabilistic, convenience, and snowball sample consisted of 393 participants. Participants for this study were eligible based on five criteria: (a) to possess a clear understanding of their decision to participate; (b) to have no history of cognitive impairments stemming from psychiatric or neurological disorders; (c) absence of substance abuse; (d) status as a family caregiver for older adults; and (e) to have experienced violent, abusive and harmful behavior inflicted by the older adult under their care.

Recruitment efforts for this study, which aimed to gather insights into cases of violent, abusive, and harmful behavior perpetrated by older individuals under the care of the participants, were executed through community channels. These methods included posting on message boards, sending private emails, utilizing list services for community centers, and social media platforms. Semi-structured interviews were conducted to gather the narratives. Participants received a comprehensive briefing about the study’s objectives and were assured that their responses would solely serve research purposes, with strict adherence to privacy.

Upon granting consent, participants were granted the flexibility to choose an online interview platform for their convenience (such as Skype, Survey Monkey, Zoom, or WhatsApp). Additionally, channels for assistance were available through phone or Internet to address any concerns. This study aims to investigate older individuals’ complex and varied abusive behaviors toward caregivers, utilizing in-depth interviews and content analysis as research methods. Through a comprehensive analysis of the various forms in which abuse presents itself, we aim to shed light on the intricate dynamics inherent in the caregiving relationship. To ensure clarity and consistency in participants’ responses, we previously provided a clear definition of harmful and abusive behavior at the beginning of the interview. The interviews primarily revolved around two central questions: “Can you describe the ways you suffered from violent, abusive, and harmful behavior by the older person for whom you care?” and “How has such behavior influenced your mental health?”

These interviews were conducted between June and October of 2022, each lasting approximately 45 min, and were scheduled once eligibility criteria and participant suitability were confirmed. Subsequently, comprehensive transcription and translation processes were undertaken to facilitate a more in-depth analysis. The research procedures adhered to ethical standards and were approved by the Research Ethics Committee at the ISPA—Instituto Universitário. These procedures were aligned with the ethical principles articulated in the 1964 Declaration of Helsinki and any subsequent updates or analogous ethical guidelines.

### Data Analysis

Following the data collection and transcription phases, an in-depth analysis was conducted using the content analysis method delineated by Erlingsson and Brysiewicz (2017). A distinctive code was attributed to each relevant category within the study, culminating in establishing a code list, which streamlined the analytical process (Erlingsson & Brysiewicz, 2017).

Three researchers individually coded all interviews. Subsequent to identifying primary themes and sub-themes, these elements were organized into coherent, separate categories that bore explicit and comprehensible labels. Through an analysis of the lived experiences and narratives of those who care for their family members, this study intended to uncover the complex emotional and psychological difficulties they encounter. Utilizing a qualitative methodology facilitated a comprehensive exploration of the subjective perspectives of individuals who provide care for family members, enabling a holistic comprehension of the emotional consequences associated with caregivers experiencing abusive behavior.

The analysis adhered to rigorous principles governing the classification and categorization of qualitative data, establishing a robust and dependable analytical system. These guidelines take into account the following factors: (a) Homogeneity: Data organization considered common elements, ensuring coherence within categories; (b) Relevance: Categories were based on their significance and importance within the data, ensuring the inclusion of meaningful information; and (c) Objectivity and fidelity: Analysis reflected a trustworthy and unbiased data representation. These guidelines were constantly followed throughout the procedure, ensuring the accuracy and reliability of the analysis.

The analysis procedure unfolded in two distinct stages: firstly, a descriptive analysis, followed by a qualitative analysis of the categories. These sequential processes culminated in creating a matrix that facilitated the interpretation of results. This systematic approach enabled an in-depth analysis and comprehensive discourse rooted in both theoretical underpinnings and empirical perspectives. The consensus between coders was robust, as evidenced by Cohen’s kappa values ranging from .85 to .93, with a *p*-value threshold of less than .01 for all analyses.

## Results

The average age of the final sample was 61.3 years (ranging from 40 to 72 years old), with a standard deviation of 5.72. Among the participants, 72.4% were women. Moreover, only 16.8% of the participants had completed high school, as indicated in Table 1. Of the participants, 88.3% lived with their partners in a relationship with the partner as the caregiver. The remaining 11.7% of participants were living alone and were cared for by first-degree family members, specifically daughters (9.4%) or siblings (2.3%).

**Table 1.** Sample of Sociodemographic and Health Characteristics.

Characteristics	Brazilian: 105 (26.7)	English: 141 (35.9)	Portuguese: 147 (37.4)	Total: 393 (100.0)
Age, average ± *SD*				*M* = 61.3 ± 5.72
Sex, *n* (%)				
Female	85 (80.5)	98 (69.5)	102 (69.4)	285 (72.4)
Male	20 (19.5)	43 (30.5)	45 (30.6)	108 (27.6)
Education, *n* (%)				
Primary school	59 (56.2)	69 (44.9)	76 (51.7)	204 (51.9)
Middle school	6 (5.7)	61 (43.3)	56 (38.1)	123 (31.3)
≥High school	40 (38.1)	11 (7.8)	15 (10.2)	66 (16.8)
Household				
Live with someone	92 (87.6)	124 (87.9)	131 (89.1)	347 (88.3)
Live alone	13 (12.4)	17 (12.1)	16 (10.9)	46 (11.7)
Family annual income, *n* (%)				
≤25,000€	46 (43.8)	61 (43.3)	63 (43.0)	170 (43.3)
>25,000€	59 (56.2)	80 (56.7)	84 (57.0)	223 (56.7)
Perceived health, *n* (%)				
Good	83 (79.0)	104 (73.8)	115 (78.2)	302 (76.8)
Poor	22 (21.0)	37 (26.2)	32 (21.8)	91 (23.2)

### Objective 1: To Explore Family Caregivers’ Experiences of Violent, Abusive, and Harmful Behavior by the Older Person

The first objective of this study aimed to explore family carers’ experiences of violent, abusive, and harmful behavior exhibited by older individuals. Six themes emerged from the analysis, including frequent and extreme verbal violence (77.3%); feeling manipulated and controlled by older adults (74.7%); experiencing unpredictable illegal circumstances provoked by older adults (62.1%); experiencing damaging financial issues provoked by older adults (43.1%); experiencing physical violence (34.2.%), and experiencing sexual violence (31.1%) (see Table 2).

**Table 2.** Key Findings Summary.

Theme	%	Verbatim example
First objective		
Theme 1: Frequent and extreme verbal violence	77.3	“He constantly yelled at me for every little thing and called me names” (Emma, female, 61 years old).
Theme 2: Feeling manipulated and controlled by older adults	74.7	“He uses his disease as a way to manipulate me into doing things for him, making me feel guilty all the time” (Michael, male, 49 years old).
Theme 3: Experiencing unpredictable illegal circumstances provoked by older adults	62.1	“My wife would drive without a license, putting herself and others at risk” (Tom, female, 62 years old).
Theme 4: Experiencing damaging financial issues provoked by older adults	43.1	“My husband fell victim to a series of online scams, who were pretending to sell miracle health cures and we lost all our savings” (Linda, female, 73 years old).
Theme 5: Experiencing physical violence	34.2.	“He occasionally become aggressive, pushing and hitting me during his outbursts” (Charles, male, 47 years old).
Theme 6: Experiencing sexual violence	31.1	“My father would sometimes touch me inappropriately. These instances were deeply troubling” (Margaret, female, 49 years old).
Second objective		
Theme 1: Depression and anxiety	89.9	“I often feel anxious, and hopeless about the future. Some days it feels like I can barely cope” (Peter, male, 52 years old).
Theme 2: Anger	81.2	“I struggle with feelings of resentment and anger towards my wife” (Mark, male, 68 years old).
Theme 3: Feeling morally isolated	78.3	“I feel lonely and in conflict with my own values and beliefs” (Emily, female, 47 years old).
Theme 4: Emotional outbursts	65.1	“There are times when stress becomes overwhelming. It’s like everything exploding” (Andrew, male, 56 years old).

Verbatim quotes indicating the variety of narratives were selected to illustrate the diversity of the studied sample. All names are fictitious.

#### Theme 1: Frequent and Extreme Verbal Violence

The first theme most mentioned by the participants was frequent and extreme verbal violence (77.3%), with a significant number of respondents highlighting the distressing nature of verbal abuse within the context of older adults. This theme was mainly verbalized by Brazilian participants (64.2%). Justine verbalized, “There have been instances where my dad becomes verbally abusive, hurling insults and hurtful words towards me. Verbal aggression often escalates during moments of frustration or confusion, making it challenging to provide the necessary care and support” (Justine, female, 51 years old). Kim also said, “Witnessing my loved one engage in such behavior is emotionally draining. Now it is more frequent, and these comments do not only affect me . . . but also other family members, creating an environment where everyone is horrible shouting” (Kim, female, 45 years old).

#### Theme 2: Feeling Manipulated and Controlled by Older Adults

The second theme that emerged prominently was feeling manipulated and controlled by older adults (74.7%), with a considerable number of respondents expressing the presence of coercive dynamics within relationships involving older individuals. This theme was mostly indicated by Portuguese caregivers (62.1%). Carla mentioned, “One example that stands out is when my elderly relative consistently employs guilt-inducing tactics to influence my actions and decisions. They would make me feel responsible for their well-being, leveraging their vulnerability to exert control over my time, emotions, and choices” (Carla, female, 53 years old). Hanna also said, “They would frequently play on my emotions, making me believe that without my constant assistance, they would suffer greatly. This manipulation not only affected my personal life and well-being but also strained my relationships with other family members” (Hanna, female, 49 years old).

#### Theme 3: Experiencing Unpredictable Illegal Circumstances Provoked by Older Adults

Participants also revealed experiencing unpredictable illegal circumstances provoked by older adults (62.1%), emphasizing actual illegal activities of the care recipients, which negatively affected the caregivers. English caregivers (55.9%) mostly verbalized this theme. Annabelle verbalized, “My mom once made impulsive decisions without considering the legal consequences. She engaged in fights with her neighbors, and disregarded important legal obligations, resulting in legal disputes” (Annabelle, female, 45 years old). Diego also explained, “There was one time when my dad engaged in fraudulent activities, such as identity theft or illegal transactions, without my knowledge or consent. In the end, these actions resulted in legal repercussions, including investigations and potential legal liabilities for both him and myself as their caregiver” (Diego, male, 55 years old).

#### Theme 4: Experiencing Damaging Financial Issues Provoked by Older Adults

Furthermore, a significant proportion of respondents shared their experiences of damaging financial issues provoked by older adults (43.1%), highlighting the adverse financial impact caused by the actions of older individuals. Portuguese participants (33.1%) mostly reported this theme, followed by 30.8% of Brazilian and 27.7% of English participants. Moreover, 34% of the participants added that these experiences were related to care recipients showing signs of cognitive decline. Sarah said, “they believe they know everything and resist accepting help and guidance regarding their financial decisions. My mom insisted on managing her finances independently, unfortunately, this led to her making poor financial choices and losing money” (Sarah, female, 47 years old). Jimmy also mentioned, “My older dad was targeted by fraudulent schemes, persuaded into making poor investment decisions. They are easy targets. This led to severe financial strain for both of us and had caused immense stress, jeopardized especially his financial security” (Jimmy, male, 50 years old).

#### Theme 5: Experiencing Physical Violence

A relevant percentage of participants reported experiencing physical violence (34.2%), shedding light on the alarming prevalence of these harmful acts perpetrated by older adults. Brazilian participants (27.2%) mostly verbalized this theme. Susan explained, “There was an incident where I attempted to administer the medication as per the doctor’s instructions, but my mom vehemently resisted and became physically aggressive towards me” (Susan, female, 41 years old). Sarah said, “In my role as a caregiver for my older brother, I’ve unfortunately faced instances of physical violence from him. There have been times when he becomes agitated or confused, and he lashes out physically, hitting me or pushing me away. It’s distressing to experience such aggression from someone I love and am trying to care for” (Sarah, female, 55 years old).

#### Theme 6: Experiencing Sexual Violence

This theme was mentioned by 31.1% of participants, and it unveiled the distressing prevalence and profound impact of sexual violence perpetrated by older adults. Brazilian participants (21.0%) mostly verbalized this theme. Lily verbalized, “I take care of my father but, sadly, I have encountered unwanted sexual advances and inappropriate behavior, leaving me feeling violated. He makes sexual comments and has even groped me once. Sometimes it’s uncomfortable to be around him” (Lily, female, 47 years old). Carla shared, “While caring for my older father, I’ve unfortunately experienced instances of sexual harassment from him. He frequently makes lewd comments about my appearance and has even made explicit remarks about specific parts of my body. It’s disheartening when caregiving takes a toll on one’s sense of safety” (Carla, female, 53 years old).

### Objective 2: To Explore How Violent, Abusive, and Harmful Behavior by the Older Person Affects Family Caregivers’ Mental Health

The second objective of this study aimed to explore how older peoples’ violent, abusive, and harmful behavior affects family carers’ mental health. Through the analysis, four themes emerged, including depression and anxiety (89.9%), anger (81.2%), feeling morally isolated (78.3%), and emotional outbursts (65.1%; see Table 2).

#### Theme 1: Depression and Anxiety

The first theme, highlighted by a significant 89.9% of older adults, focused on depression and anxiety felt by family caregivers. This theme was the most reported by English participants (84.3%). Mental health issues underscore the substantial impact of violent, abusive, and harmful behavior on the mental well-being of caregivers. Sophie mentioned, “Their unpredictable and sometimes violent behavior adds an additional layer of complexity to the caregiving process. This kind of behavior intensifies my anxiety, as I constantly worry about the safety of both him and myself, and even other people present, like his grandchildren” (Sophie, female, 42 years old). Eve, who had a history of depression, also verbalized, “Their challenging behaviors, declining health, and the weight of responsibility exacerbate my own feelings of depression. Witnessing their physical and cognitive decline, along with the emotional toll of managing their daily needs, can trigger a sense of sadness, hopelessness, and emotional exhaustion” (Eve, female, 49 years old).

#### Theme 2: Anger

The next theme, reported by 81.2% of respondents, centered around anger. This theme was mostly referred to by Brazilian caregivers (76.3%). This prevalence highlights the considerable presence of this intense emotional response among family caregivers. Eric said, “These emotionally abusive behaviors create a toxic and hostile environment, eroding my self-esteem and mental well-being. It makes me angry because I try to help, and I still have to go through this? I think it’s unfair . . .” (Eric, male, 41 years old). Philip also mentioned, “I feel angry and frustrated that I can’t provide them with the care they need without endangering myself or others. It’s unfair that some have to go through this and others [family members] don’t” (Philip, male, 49 years old).

#### Theme 3: Feeling Morally Isolated

The subsequent theme was feeling morally isolated, as 78.3% of participants noted, revealing the intricate link between older individuals’ challenging behavior and its profound psychological impact on caregivers. Portuguese participants (66.8%) mostly verbalized this theme. Diana verbalized, “Sometimes her behavior is not ideal, she has had aggressive outbursts, physically hurt herself or others, or exhibited verbally abusive behavior. These actions go against my moral compass and create a deep sense of isolation as I find it difficult to reconcile their behavior with the compassionate caregiver role I aspire to play” (Diana, female, 43 years old). Thomas said, “she tries to take advantage of unsuspecting family members or acquaintances for personal gain. Witnessing the harm caused by her deceitful actions leaves me morally conflicted and isolated, as I struggle to reconcile their behavior with my own ethical values” (Thomas, male, 54 years old).

#### Theme 4: Emotional Outbursts

Reported by 65.1% of participants, the subsequent theme delves into “Emotional Outbursts,” highlighting the significant occurrence of these intense emotional reactions among family caregivers. This theme was mostly verbalized by English participants (54.7%). Olivia explained, “When they engage in verbal or physical aggression, resulting in heightened emotions of anger, frustration, and even helplessness. The constant stress of managing their outbursts takes a toll on my own emotional well-being, leading to my own occasional emotional outbursts” (Olivia, female, 51 years old). Sean mentioned, “she becomes verbally abusive, making hurtful remarks and demanding constant attention. While I understand that it’s not her fault for needing help, there are times when I feel she may not fully understand that it’s hard for me too. And sometimes our conversations end with emotional outbursts” (Sean, male, 48 years old).

## Discussion

This qualitative research study explores the complex dynamics of abusive behaviors exhibited by senior care receivers against their family carers. The present study is characterized by a dual focus, comprising an in-depth analysis of the diverse forms of abuse seen by carers and a thorough understanding of the subsequent effects on their psychological well-being. The findings further underscore the intricate and multifaceted nature of these relationships, highlighting the complex interplay of factors influencing caregiver-care recipient dynamics in situations of violent, abusive, and harmful behavior perpetrated by the older person toward their family caregivers. As a pioneering endeavor in its field, this study sheds light on several facets of abuse, providing a thorough depiction that enhances the ongoing academic conversation.

The participants reported a wide range of abusive behaviors shown by older care recipients toward them. Abuse within the caregiver-care-recipient dynamic presents a multifaceted health and social dilemma within family settings (Välimäki et al., 2020). The high occurrence of narratives provided by the participants serves as evidence of a concerning situation that encompasses instances of frequent and extreme verbal violence (77.3%), feeling manipulated and controlled by older adults (74.7%), experiencing unpredictable illegal circumstances provoked by older adults (62.1%); experiencing damaging financial issues provoked by older adults (43.1%); experiencing physical violence (34.2.%); and experiencing sexual violence (31.1%).

This comprehensive depiction of abuse presents an unusual perspective, as previous studies in the literature have primarily focused on quantifying the frequency and occurrence of caregivers experiencing abusive behavior. Previous research on abuse categories has been limited, with only a few studies, such as those conducted by Paveza et al. (1992), Steinmetz (1988), and Wang et al. (2019), addressing this topic.

Previous research on cultural differences in the relationship between caregivers and care recipients is still insufficient (Isham et al., 2019; Pinyopornpanish et al., 2022). Moreover, variations arose among different cultures when delving into the most frequent themes. Brazilian caregivers mainly reported the first theme, while Portuguese mostly verbalized feeling manipulated and controlled by older adults. Moreover, experiencing unpredictable illegal circumstances provoked by older adults was mostly verbalized by English participants, therefore showing legal uncertainties as a pertinent concern. Portuguese participants shed light on experiencing damaging financial issues provoked by older adults, reflecting the significance of economic concerns in their context. Last, experiencing physical violence and sexual violence were both mostly verbalized by Brazilian participants, highlighting the frequent experience of violence by family caregivers. Cultural norms shape views on family roles and the caring of older people. For instance, in some cultures, there are cultural traditions of reverence for older people, making it difficult for caregivers to report abuse. In other cultures, abuse by care recipients may be considered a private matter, discouraging disclosure and intervention. Recognizing these cultural nuances is essential for crafting effective interventions to address abuse by care recipients across diverse communities (NetCE, 2023; von Humboldt, Monteiro, & Leal, 2018).

Frequent and extreme verbal violence was the most verbalized theme by these participants. In fact, in Steinmetz’s (1988) investigation, it was found that a notable proportion of caregivers, namely 23%, suffered from acts of violence from older individuals under their care. Additionally, a higher percentage of older adults, precisely 34%, exhibited verbal abuse, including false accusations, while 18% displayed physical aggression. The study by Hamel et al. (1990) also examined a sample of 213 family carers responsible for caring for older individuals. According to their findings, a majority of 57.2% of respondents reported experiencing verbal aggression from older individuals. The results indicate that verbal aggressiveness was the most prevalent form of aggression, accounting for 51% of reported incidents.

Furthermore, physical aggression was reported by 34% of participants, while sexual hostility was reported by 31.1% of participants. Moreover, Phillips et al. (2000) observed that around 29% of the 93 individuals serving as caregivers had instances of verbal abuse while providing care to older adults. Current findings indicate that participants reported instances of verbal maltreatment, namely false accusations perpetrated by dependent older adults. Findings from Ayres and Woodtli (2001) and von Humboldt Leal, Laneiro, et al. (2013), von Humboldt & Leal (2014b), (2017) indicated that caregivers with past trauma were more likely to engage in the mistreatment of the care recipient, which can include various forms of abuse or neglect, as well as making false statements, show reciprocal violence, and self-defense behaviors. This tendency is particularly pronounced in caregiving situations involving care recipients with cognitive decline, adding further complexity to the caregiver-care-recipient relationship.

The second most mentioned theme was feeling manipulated and controlled by older adults, mainly guilt-inducing tactics and emotional manipulation. The first involved older adults making caregivers feel responsible for their well-being, exploiting their vulnerability to influence their actions. In contrast, the second entailed care recipients frequently playing on caregivers’ emotions. Ayres and Woodtli (2001) also delve into the intricacies of control dynamics between older patients and their carers by examining various strategies employed by older patients, such as manipulation, abusive controlling, pouting, violation of privacy, physical abuse, rejection of medication, and involvement of law enforcement. Moreover, older adults in cognitive decline may report abusive behavior by a caregiver that did not occur; however, care recipients with dementia are especially vulnerable to maltreatment (Ayres & Woodtli, 2001). The findings of their study underscore the importance of recognizing that older caregivers may face risks due to the violence exhibited by the older patients under their care.

The next theme indicated by participants was experiencing unpredictable illegal circumstances provoked by older adults. These accounts highlight the often-unforeseen legal challenges that can arise within caregiving dynamics, emphasizing the importance of a well-informed and adaptable approach for both caregivers and older individuals. When old age and illness coincide, legal considerations take on particular importance. Family members are crucial as caretakers as older adults’ health declines and frailty threatens their independence. While some families prepare for the many financial, legal, and personal issues that come with providing care, these talks are usually delayed until the need is pressing (Schumacher et al., 2006).

These participants also verbalized their experience of damaging financial issues provoked by older adults, shedding light on the caregivers’ multifaceted challenges in their caregiving roles. Equally intriguing are the narratives that elucidate the complex ramifications of financial upheaval generated by the activities of care recipients. Concerning this, the insightful point made by Hansen et al. (2020) regarding the possibility of property damage caused by older adults as a triggering factor for economic instability holds significant resonance. Moreover, 34% of the participants in our study indicated that these experiences were related to care recipients who were showing signs of cognitive decline, which corroborates existing literature on violence directed at caregivers by individuals with cognitive decline and dementia (Neha et al., 2021; Wildman et al., 2023).

Among these participants, the experience of physical violence was frequently reported. According to Åström et al. (2004), caregivers may also receive injuries such as wounds and bruises. Numerous research papers provide empirical evidence supporting the assertion that abuse is a prevalent phenomenon experienced by persons responsible for caring for patients with disabilities or chronic medical issues, including older adults. Nevertheless, there is a dearth of research studies that specifically examine the psychological ramifications resulting from the abusive conduct directed toward those in the role of caretakers by older care recipients. According to a study by O’Leary et al. (2005), 20% of individuals diagnosed with AD exhibited physical violence toward their caretakers. This aggressive behavior was more prevalent in the middle stage of AD than in the early stage.

Furthermore, caregivers experiencing patients with aggressive behavior, such as those diagnosed with AD and exhibiting severe behavioral issues, were found to be correlated with negative impacts on caregivers’ mental health (Vespa et al., 2021). Moreover, research documents instances of caregivers being subjected to abuse by older persons who are afflicted with mental health disorders such as schizophrenia and psychosis. A study conducted by Wang et al. (2019) found that a significant proportion (70.3%) of primary carers for individuals with severe mental health diseases reported verbal and physical abuse perpetrated by the patients. Furthermore, emotional instability and anxiety during life-changing experiences, such as the onset of cognitive decline or other health conditions, may indirectly lead to aggression (Mitchell et al., 2011).

The study conducted by Coyne et al. (1993) examined the phenomenon of caregivers experiencing abusive behavior among a cohort of 342 carers responsible for individuals diagnosed with dementia. A notable proportion of caregivers, specifically 12%, acknowledged resorting to physical violence while fulfilling their caregiving responsibilities. Conversely, a far higher percentage of caregivers, totaling 33%, reported occurrences where the older individuals under their care exhibited aggressive behavior toward them. Cahill and Shapiro (1993) documented comparable findings in their investigation of aggressiveness exhibited by dementia patients toward their caretakers. Specifically, they found that 89% of female caregivers reported experiencing violent conduct from their dependent patients. In relation to this, the participants of our study did not report reciprocal violence within caregiver-care-recipient relationships, since they did not retaliate against any violence from the care recipient.

The last theme mentioned by the participants was the experience of sexual violence. While sexual abuse disclosures are reported, there has been little focus on family caregivers enduring sexual violence from the older adults they care for (Hughesy, 1997; Phillips et al., 2000). This distressing scenario arises when caregivers face repeated instances of sexual violence, leading to feelings of manipulation and control (Hughesy, 1997; Phillips et al., 2000) and it was initially discussed by Hamel et al. (1990), who reported that ultimately, sexual behavior played a pivotal role in determining whether home care services would be discontinued. Indeed, sexual violence adds complexity to family dynamics, where the interplay of power dynamics, familial ties, and obligations becomes deeply intertwined (Hughesy, 1997; Phillips et al., 2000).

Regarding the second objective, the impact of such maltreatment on caregivers’ mental health is elucidated with striking clarity. Providing care entails a complicated web of emotional intricacies (Kokorelias et al., 2019). The individuals involved in this study vividly describe various mental health challenges. Depression and anxiety (89.9%), anger (81.2%), moral isolation (78.3%), and emotional outbursts (65.1%) can increase the caregivers’ distress and compromise their well-being. When examining the broader scope of literature, notable similarities become apparent, emphasizing the significant influence of this phenomenon. The studies conducted by VandeWeerd et al. (2013), Graneheim et al. (2012), and Wharton and Ford (2014) align with and support the conclusions of the present investigation. Additionally, research by Erosa et al. (2010), Åström et al. (2004), Gates et al. (1999), Pinyopornpanish et al. (2022), and Vespa et al. (2021) further corroborate the negative consequences highlighted above, thereby enriching the comprehension of the emotional challenges experienced by caregivers and fostering a collective understanding of shared experiences.

Variations emerged among different cultures when exploring the themes for the second objective. English caregivers mostly reported depression and anxiety while anger was mostly verbalized by Brazilian participants. Portuguese caregivers and English participants mostly indicated moral isolation and emotional outbursts. These findings corroborate existing research (Bhugra et al., 2021; Pinyopornpanish et al., 2022).

The very high occurrence of narratives indicates an upsetting influence on caregivers’ mental health. Indeed, participants reported that violent, abusive, and harmful behavior by older adults contributed negatively to their mental health through depression and anxiety (89.9%), anger (81,2%), feeling morally isolated (78.3%), and emotional outbursts (65.1%).

A shared understanding also emerged across nationalities in relation to the second objective, for the most verbalized impact on their mental health: Depression, anxiety, and anger. However, differences emerged concerning the subsequent frequently raised topics.

Feeling morally isolated was reported mainly by Brazilian participants, shedding light on the moral and emotional exclusion experienced by caregivers. Additionally, English caregivers mostly verbalized emotional outbursts, revealing the complex tension resulting from intense and unpredictable reactions, as corroborated in the literature (Carmichael & Ercolani, 2016; Greenberg et al., 2020).

The first theme these participants mentioned was feeling depression and anxiety. Caregivers themselves experience emotional challenges such as feelings of inadequacy, self-disdain, diminished self-worth, and depressive symptoms (VandeWeerd et al., 2013). Erosa et al. (2010) also pointed out that in their sample, caregivers abused by care recipients experienced more significant emotional difficulties, distress, and caregiver burden than those not abused by care recipients. Moreover, abusive behavior can also lead to caregiver burnout (Gates et al., 1999) and contribute to caregiver burden and depression (Pinyopornpanish et al., 2022; Vespa et al., 2021). Numerous research such as those conducted by Neha et al. (2021), Wildman et al. (2023), and Labrum et al. (2021), have examined incidents of violence directed at caregivers by individuals with severe mental health disorders.

Feelings of anger were the next theme verbalized by these participants. Violence directed at caregivers in nursing homes elicits various emotional responses, such as aggressiveness, shock, aversion, or fear (Erosa et al., 2010). Moreover, VandeWeerd et al. (2013) suggested that intense aggression or violence within caregiving contexts leads to adverse consequences for families, including distress, compromised life functioning, and cognitive impairments. According to a study by Graneheim et al. (2012), carers who interacted with care recipients exhibiting violent or aggressive behavior had heightened vigilance and a heightened sense of revulsion toward such behavior. As a result, these caregivers expressed disappointment, helplessness, and a sense of personal failure due to their inability to handle challenging behavior effectively (Wharton & Ford, 2014).

In their research conducted in 1992, Paveza and his colleagues examined the correlation between severe family violence and dementia in a sample of 184 older individuals residing in the community. These individuals were part of a patient registry centered on AD. The results revealed that a significant portion of caregivers, specifically 16%, reported instances of severe physical aggression exhibited by the older individuals they were caring for. According to Pillemer and Suitor’s (1992) study, caregivers who have encountered violence from older care recipients are more at risk for future victimization. This finding implies that older care recipient can potentially instigate more violence toward their caregivers.

Moreover, an alarming pattern emerges as scholarly studies illuminate cases where caregivers are unjustly accused of abuse and neglect, as shown by Snyder et al. (2015). Participants in this study also pointed out feeling morally isolated. Research consistently indicates that providing care for individuals with complex needs is associated with heightened risks to both physical and mental well-being, increased levels of social isolation, and precarious or unsatisfactory employment situations (Carmichael & Ercolani, 2016). In the Bonin-Guillaume et al. (2022) study, 10% of the 876 family carers reported feeling lonely regularly. In addition to being solely in charge of caring for their parents, caregivers who reported loneliness were twice as likely to be dealing with a heavy caregiving burden. Compared to individuals who did not experience such feelings of loneliness, this group saw a higher prevalence of physical and mental health issues. The likelihood that individuals would have anxiety, be in fragile physical condition, struggle with sleep issues, and think poorly of their general health was also raised.

The last theme indicated by the participants was emotional outbursts. Onwumere et al. (2018) conducted a systematic review to evaluate the effects of patient bursts of violence on caregivers, shedding light on the significant impact such behavior can have on caregivers’ well-being and mental health. The review identified several consequences of violent behavior toward caregivers, including emotional distress, intrusive thoughts, avoidance, hyperarousal symptoms, fear of future violent incidents, perception of life-threatening situations, feelings of frustration, powerlessness, and inability to manage the patient’s behavior. Additionally, caregivers reported a deterioration in their relationship with the patient. Nevertheless, the age range of the patients was not specified in this study. Higher-hour caregivers are also prone to experiencing emotional distress, financial hardship, and physical strain. As a result of providing high-hour care services, these caregivers are likely to be in fair or poor health due to balancing increased levels of housework and facing challenges in coordinating with healthcare professionals (Greenberg et al., 2020). Ensuring the safety of caregivers and older adults is a priority. However, due to the prominence of teleworking and unemployment, the availability of respite care has decreased. Finding necessary pauses and personal space is complex because of few choices. This issue is crucial for people who care for dementia patients at home, especially those who are prone to violent conduct or emotional outbursts (Greenberg et al., 2020).

This study has some limitations. Significantly, the study’s qualitative approach exclusively examined caregivers who had experienced abuse, neglecting to incorporate the viewpoint of carers who had not experienced abuse. Conducting a broad examination that spans both groups would provide a more comprehensive understanding of the specific effects of abusive conduct on mental health. Future study initiatives could explore the intricate variations of abusive behaviors demonstrated by older individuals who experience diverse diseases, including dementia, stroke, chronic pain, disability, or general symptoms associated with aging. This customized strategy can potentially enhance the effectiveness of targeted interventions and educational programs, as they are designed to address the distinct obstacles associated with particular health conditions specifically.

Another difficulty or limitation we encountered while designing this research is the lack of a comprehensive definition of this construct. A specific definition, theoretical framework, and possible dimensions and factors of this construct can help us understand and study it better. Moreover, while efforts were made to provide a clear definition of harmful and abusive behavior to participants, variations in individual interpretations may have influenced the reporting of experiences. Also, longitudinal research studies can help us understand the patterns of caring, conflict, and abuse among family members. Furthermore, another limitation of this study is the fact that reciprocal violence within caregiver-care-recipient relationships was not a topic of discussion due to the protocol employed, which suggests the need for future research in this area.

Moreover, considering that 88.7% of the participants were partners living with the care recipient and the remaining 11.3% were first-degree family members, specifically daughters (9.4%) or siblings (2.3%). living with the care recipient, the familial relationship between the caregiver and the care recipient varied among participants. This suggests that more attention may need to be given to how different types of relationships, such as parent/child versus spousal relationships, influence the dynamics of abuse by care recipients. The scope of the future investigation can encompass the influence of the family relation of the recipient with the caregiver, caregivers’ personality qualities, self-esteem, and social support on the expression of abusive behaviors and the following strategies for managing them. By comprehensively understanding these influential elements, we may enhance our ability to develop informed approaches for prevention and support. Moreover, future research may deepen the underlying motivations behind the recipients’ behavior toward the caregiver, which can provide valuable insights into the dynamics of complex caregiving relationships. Moreover, since the literature on sexual violence toward family caregivers is still scarce, future studies may address this crucial topic.

Notwithstanding these limitations, this pioneering research represents a unique and original investigation, focusing on the mental health of informal caregivers who experience mistreatment from older care recipients. In contrast to prior scholarly investigations that predominantly focused on documenting the prevalence of caregivers experiencing abusive behavior and delving into the underlying motivations behind abusive conduct exhibited by carers, the present study represents a significant advancement in the field, as it introduces a novel avenue for inquiry that has not been previously explored.

The ramifications of the findings from this research study are of great importance for healthcare professionals and stakeholders. A focus on older adults’ dependency, as well as caregiver burden, has recognized the need to look at the complexity of the caregiver and care recipient relationship in explaining abuse/neglect. The insights provided serve as a significant tool for recognizing patterns of abuse and neglect within dynamics involving older individuals, with caregivers playing a pivotal role as critical sources of information. Future studies should further explore the complexity of the caregiving relationship, particularly when addressing maltreatment and negligence. Moreover, these findings highlight the diversity of experiences within different cultures. They may enrich educational initiatives and interventions that enable caregivers from different cultural backgrounds to address and successfully manage abusive behaviors while safeguarding their own welfare and fostering healthier caregiving relationships.

This ground-breaking research study functions as a catalyst for subsequent investigation and intervention. The insights presented emphasize the necessity for continuous investigation and the establishment of customized support systems. As the scope of research broadens, the findings of this study will undoubtedly enhance our comprehension, ultimately facilitating a more empathetic and knowledgeable approach to addressing abuse within caregiving relationships. By recognizing the distinct themes identified, policymakers and healthcare professionals can develop specific interventions and support techniques to enhance the emotional well-being and shield the mental health of family caregivers who are confronted with abusive behaviors from older individuals. Moreover, considering that the motivations for violence from care recipients with cognitive decline are different from other care recipients, interventions and policy programs can comprise specialized training and support for caregivers, as a way of promoting safety and well-being for both parties.

In conclusion, this study highlights the imperative to acknowledge the frequently veiled yet unmistakable occurrence of abusive actions exhibited by older care users against their carers. This research delves into the various forms of abuse and their significant impact on mental health, presenting findings that extend beyond the confines of scholarly investigation. The study’s extensive ramifications contribute to the academic field and prompt society to respond promptly and empathetically. The study’s findings carry significant consequences for healthcare practitioners, governments, and those with a vested interest in the matter. The imperative to educate carers, develop interventions customized to their needs, and establish comprehensive support systems arises to empower caregivers with effective coping skills and enable them to negotiate the hurdles presented by abusive behaviors.

In brief, this qualitative study explored the complex dynamics of abusive actions demonstrated by older care users against their carers, providing insight into a frequently neglected aspect. Employing persistent investigation and focused interventions, the scope of this study can develop into a complete depiction that provides insights for policies and initiatives, ultimately mitigating the impact of abuse on carers, promoting mental health, and fostering a compassionate caregiving environment.
